# Bilateral retinitis following typhoid fever

**DOI:** 10.1186/s40942-017-0065-z

**Published:** 2017-04-10

**Authors:** M. Prabhushanker, Tasneem T. Topiwalla, Geetha Ganesan, Sripal Appandaraj

**Affiliations:** Sankara Eye Centre, Sathy Road, Sivanandapuram, Coimbatore, 641035 India

**Keywords:** Post typhoid fever, Immune mediated, Typhoid retinopathy, Retinitis

## Abstract

**Background:**

Post typhoid fever immune related reactions affecting the eye is a rare finding which can have various presentations in which typhoid retinopathy is not a well recognized sequelae.

**Case presentation:**

Here we present a case of 59 year old male who presented with right eye sudden painless loss of vision 4 weeks after typhoid fever which was diagnosed and treated successfully. His BCVA was 2/60 in right eye and 6/6 in left eye. Fundus examination showed retinitis along with macular serous detachment in right eye and retinitis in left eye. Significant improvement in BCVA in right eye was observed after treatment with oral steroid with resolving retinitis lesions. Diagnosis of post typhoid immune mediated retinitis was made with good resolution following treatment.

**Conclusions:**

Immune mediated retinitis is a rare sequelae to typhoid infection which can be successfully treated with systemic steroids with good resolution of the lesions.

## Background

Typhoid or enteric fever is a systemic disease which is characterized by fever and abdominal pain caused due to dissemination of *Salmonella typhi* or *paratyphi*. It is transmitted by food or water due to fecal contamination by ill or asymptomatic chronic carriers. A high incidence of typhoid fever in developing countries correlates with poor sanitation and lack of access to clean drinking water [1]. Ocular manifestations of typhoid fever are rare and include lid edema or abscess, dacryoadenitis, conjunctival petechiae or chemosis, corneal ulceration, uveitis, vitreous haemorrhage, retinal haemorrhage and detachment, stellate maculopathy, pigmentary retinopathy, optic neuritis, internal or external ophthalmoplegia, orbital haemorrhage or abscess. These complications are caused either by direct invasion of the organisms into the ocular tissue, or by hypersensitivity reaction such as vitreous haemorrhage after typhoid vaccination [2]. Here we are presenting a case of retinitis with macular serous detachment developing post typhoid fever.

## Case report

**Fig. 1:**
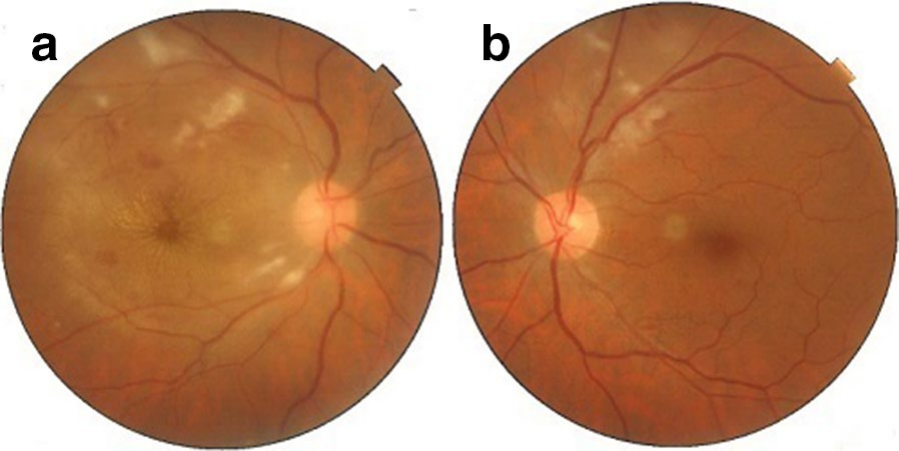
**a** Fundus *photo of right eye* with white fluffy lesions suggestive of retinitis in the superior and inferior temporal arcades with macular star. **b** Fundus *photo of left eye* with white fluffy retinitis lesions in the superior temporal arcade

**Fig. 2:**
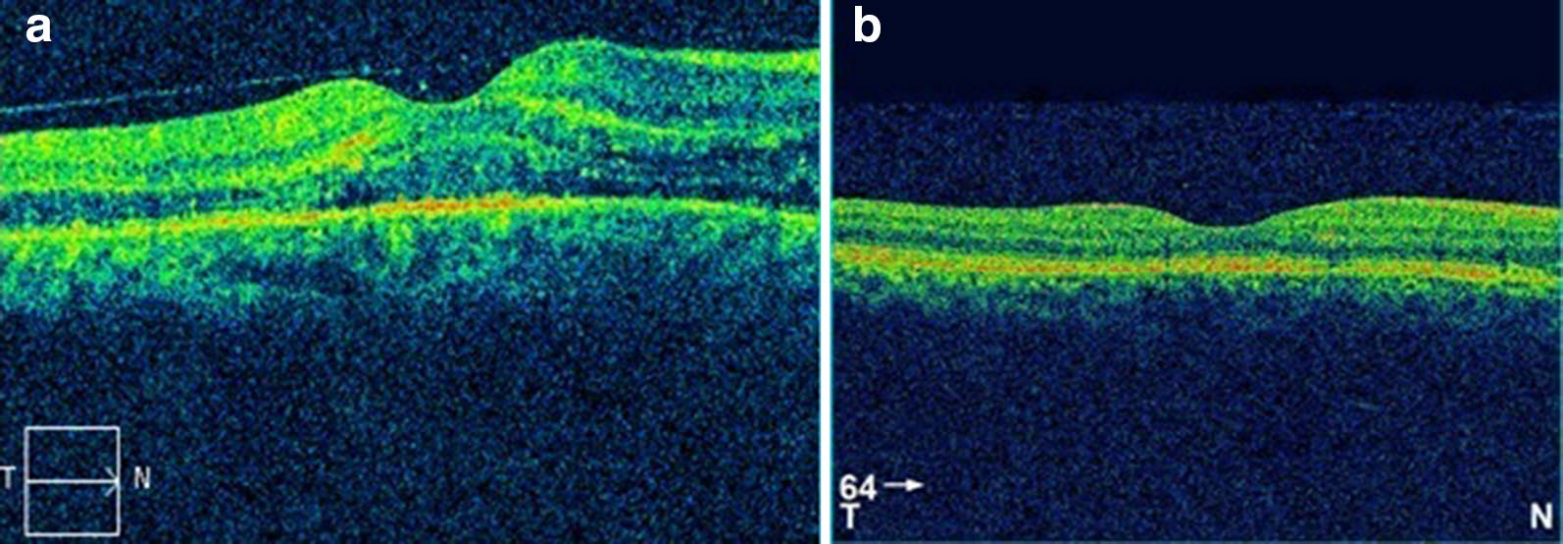
**a** Pre treatment OCT of *right eye* suggestive of macular edema. **b** A normal OCT *photo of right eye* post treatment

**Fig. 3:**
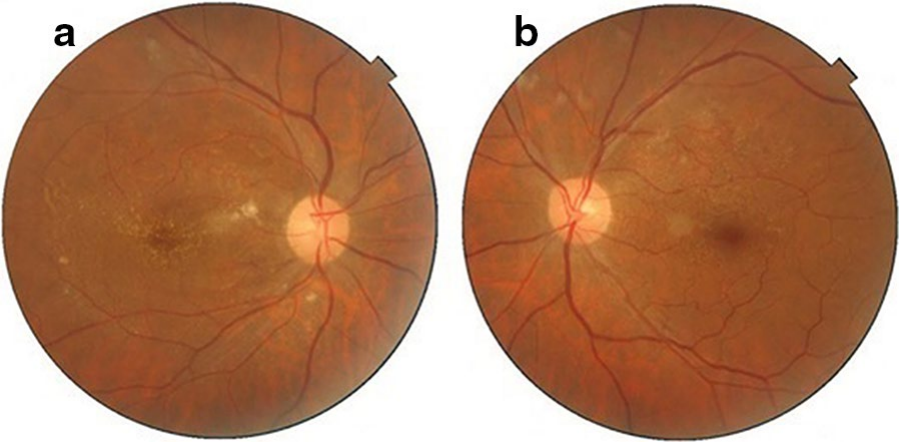
**a** Post treatment fundus *photo of right eye* showing resolved retinitis lesions with few dispersed hard exudates. **b** Post treatment fundus *photo of left eye* with resolved retinitis lesions

A 59 year old male presented to our hospital 1 week after experiencing diminution of vision in the right eye. He gave a history of typhoid fever 4 weeks prior to presentation for which Widal test was performed to confirm diagnosis. The test results showed significant titres for ‘O’ antigen (>1:80) and ‘H’ antigen (>1:160) and negative for ‘AH’ and ‘BH’ antigens. He was subsequently started on oral Ofloxacin 400 mg twice daily for 2 weeks following which fever subsided. There was no known history of diabetes mellitus or hypertension. On ocular examination his best corrected visual acuity was 2/60 in the right eye and 6/6 in left eye. Anterior segment findings were unremarkable with IOP being within normal range for both eyes. Fundus examination of right eye showed white fluffy lesions along the superior and inferior arcades with superficial haemorrhages in around the macula with a macular star suggestive of retinitis (Fig. 1a). Left eye fundus showed few dispersed retinitis lesions with superficial haemorrhage along the superior arcade with intact foveal reflex (Fig. 1b). On optical coherence tomography of right eye underlying macular serous retinal detachment was noted (Fig. 2a). Blood tests were done to rule out VDRL and HIV status. X-cyton analysis of the anterior chamber aspirate was negative for organisms like Mycobacterial Tuberculosis, Toxoplasma Gondii, Hepes Simplex Virus, Cytomegalovirus and Varicella Zoster Virus. After analysis of the reports diagnosis of post typhoid retinitis in both eyes was made. Patient was started on oral prednisolone 1 mg/kg body weight which was tapered over 2 months along with monitoring of systemic and ocular health. Patient came for follow up every 2 weeks for 3 months. Every visit fundus photo was documented. After 2 months of initiating treatment there was an improvement in the BCVA in right eye to 6/6 which was maintained on further visits. Fundus examination revealed resolving lesions in both eyes (Fig. 3a, b) and OCT of the right eye showed resolution of the serous detachment (Fig. 2b).

Retinitis is characterized by confluent areas of retinal whitening which progresses along the retinal blood vessels, often associated with intraretinal hemorrhages and hard exudates. A significant number of retinitis cases are thought to be idiopathic in etiology but a small proportion of cases are infectious in etiology such as *Toxoplasma gondii* (toxoplasmosis), *Leptospira* spp. (leptospirosis), *Mycobacterium tuberculosis* (tuberculosis) and other viral and fungal etiologies [3]. Non infectious causes of retinitis include sarcoidosis, Behcet’s disease. Infectious causes are usually unilateral and may be associated with mild vitritis. Patients can present with Neuroretinitis like picture with optic disc edema and macular hard exudates [3]. The macular star becomes prominent over first 3 weeks with neuroretinitis resolving over 6–8 weeks [4]. Leakage from the optic nerve head can lead to retinal swelling, exudation and edema, whereas retinal venous occlusion due to vasculitis results in intraretinal haemorrhage, cotton wool spots and retinal and optic nerve head edema [5]. It was postulated that microbial pathogens may be responsible for immune mediated ocular and systemic pathology through postinfectious immunological effects. These may be due to molecular mimicry eliciting an immune response that cross react with self antigens. Even though active infection is an unusual cause of retinal vasculitis, it is possible that many idiopathic and systemic disease associated cases are precipitated by previous encounters with microbes bearing DNA sequence homologous to retinal and vascular autoantigens. Similarity between S-antigen and peptides derived from yeast, *E. coli*, and Hepatitis B virus was found and there was an ability of these microbial peptides to elicit an immune response post infection [5]. Immune mediated retinitis is a clinical diagnosis most often when there is past history of infection few weeks or days prior to the onset of ocular manifestations. In this case, treatment with oral steroids was initiated due to inflammation of the retina, especially the macula which caused decrease in vision. By taking into consideration the time of onset of ocular presentation, previous history of typhoid fever and the response to oral steroids; the most likely diagnosis was post typhoid fever immune mediated retinitis with macular neurosensory detachment in the right eye and retinitis in left eye. In our case as the disc edema was not prominent, neuroretinitis was not considered as the diagnosis. Similar case reports by Relhan et al. [6] and Laul et al. [7] showed immune mediated response post typhoid fever presenting with neuroretinitis, vasculitis and macular detachment. Successful treatment with steroids was seen in them. Fusco et al. [8] reported a case of bilateral chorioretinitis and stellate maculopathy post typhoid fever. However, in our case Xcyton analysis was done to rule out possible infectious retinitis before initiating steroid therapy, as it could exacerbate non immune mediated retinitis. Xcyton multiplex PCR analysis even though has the advantage of increasing the diagnostic yield it has certain disadvantages like false positive and negative results due to cross reactivity and preferential amplification, negative internal control if there is high amount of a particular target causing exhaustion of reagents and high cost.

## Conclusion

Immune mediated retinitis can occur following systemic infection and can be managed with steroids followed by good resolution of the lesions. PCR analysis of the aqueous is a rapid diagnostic tool wherein multiple organisms can be detected and sight threatening bacterial and viral infections can be ruled out before initiation of steroid therapy.
